# Delivery of Topically Applied Calpain Inhibitory Peptide to the Posterior Segment of the Rat Eye

**DOI:** 10.1371/journal.pone.0130986

**Published:** 2015-06-24

**Authors:** Taku Ozaki, Mitsuru Nakazawa, Tetsuro Yamashita, Sei-ichi Ishiguro

**Affiliations:** 1 Department of Ophthalmology, Hirosaki University Graduate School of Medicine, Hirosaki, Japan; 2 Department of Biological Chemistry, Iwate University Faculty of Agriculture, Morioka, Japan; 3 Department of Biochemistry and Molecular Biology, Hirosaki University Faculty of Agriculture and Life Science, Hirosaki, Japan; Univeristy of Miami, UNITED STATES

## Abstract

We developed an inhibitory peptide that specifically acts against mitochondrial μ-calpain (Tat-μCL, 23 amino acid, 2857.37 Da) and protects photoreceptors in retinal dystrophic rats. In the present study, we topically administered Tat-μCL to the eyes of Sprague-Dawley rats for 7 days to determine both the delivery route of the peptide to the posterior segment of the eye and the kinetics after topical application in adult rats. Distribution of the peptide was determined by immunohistochemical analysis, and enzyme-linked immune-absorbent assay was used to quantify the accumulation in the retina. Peptides were prominently detected in both the anterior and posterior segments of the eye at 1 h after the final eye drop application. Immunohistochemically positive reactions were observed in the retina, optic nerve, choroid, sclera and the retrobulbar tissues, even in the posterior portion of the eye. Immunoactivities gradually diminished at 3 and 6 h after the final eye drop. Quantitative estimations of the amount of peptide in the retina were 15.3, 5.8 and 1.0 pg/μg protein at 1, 3 and 6 h after the final instillation, respectively. Current results suggest that while the topically applied Tat-μCL peptide reaches the posterior segment of the retina and the optic nerve, the sufficient concentration (> IC50) is maintained for at least 6 h in the rat retina. Our findings suggest that delivery of topically applied peptide to the posterior segment and optic nerve occurs through the conjunctiva, periocular connective tissue, sclera and optic nerve sheath.

## Introduction

Although suggestions that topically instilled peptides are delivered to the posterior segment ocular tissues has been controversial [1], previous studies have demonstrated that some peptides do reach the retina after topical application [2–5]. Moreover, it has been shown that conjunctivally instilled insulin (5,000 Da) [2], nerve growth factor (26k Da) [3] and antibody fragments (26 and 28k Da) [4, 5] can penetrate either rat or rabbit eyes, with accumulation in the retina and optic nerve observed to some extent for hours. From a clinical standpoint, topical application of therapeutic agents to the posterior segment of the eye is more advantageous compared to direct intraocular injection or systemic administration in terms of simplicity, safety and patient convenience. We previously developed an inhibitory peptide against rat mitochondrial μ-calpain [6, 7]. This calpain subgroup has been known to play an important role in photoreceptor cell death in the dystrophic rat retina [8]. In addition, we also demonstrated that topical application of this mitochondrial μ-calpain inhibitory peptide resulted in protective effects against photoreceptor degeneration [6, 7]. The peptide consists of a total of 23 amino acids (2857.37 Da) with a 10 amino acid stretch of the μ-calpain inhibitory domain at the carboxy-terminal end and an additional 13 amino acid fragment of the human immunodeficiency virus (HIV) Tat peptide conjugated at the amino-terminal end in order to enhance membrane penetration (Tat- μCL). Based on previously reported results that showed topically applied insulin could reach the rat retina [2], we further examined the application of Tat- μCL eye drops to the eyes of Royal College Surgeon’s (RCS) rats [6], in addition to evaluating the topical instillation of the eye drops in rhodopsin transgenic S334ter and P23H rats [7]. However, the specific delivery routes and the bio-turnover of relatively low molecular weight peptides like Tat- μCL remain unknown in the eye. This information is both important and necessary if a proper clinical protocol for topical therapy using Tat- μCL is to be designed in the future. Therefore, our present study performed immunohistochemical and biochemical analyses to clarify the pharmacokinetics and distribution of topically instilled Tat- μCL in the adult rat eyes.

## Materials and Methods

### Animals

All experimental procedures were designed to conform to the Association for Research in Vision and Ophthalmology (ARVO) Statement for the Use of Animals in Ophthalmic Vision Research. The study protocol was also approved by the Committee for the Use of Live Animals at the Hirosaki University (Permit Number: M10016). Sprague-Dawley (SD) rats were purchased from Clea Japan (Tokyo, Japan) and maintained at the Hirosaki University Graduate School Animal Care Service Facility. The rats were housed three rats to a cage with ad-libitum access to food and water under a 12-h light (50 lux illumination) and 12-h dark (<10 lux illumination) cycle with a temperature controlled environment and SPF condition. Care was taken not to cause photoreceptor light damage in the rats.

### Synthesis of Tat- μCL

Tat-μCL (amino acid sequence, GRKKRRQRRRPPQPDALKSRTLR) was synthesized in accordance with our previously reported method [6, 7]. Briefly, the peptide was synthesized by the fluorenylmethyloxycarbonyl method using an automated peptide synthesizer (Shimadzu PSSM-8; Shimadzu, Kyoto, Japan). The synthesized peptide was then purified by reverse-phase high performance liquid chromatography using a C18 column (Jupiter 250 mm × 10 mm; Phenomenex, Torrance, CA). The molecular weight and purity (>95%) of the peptide were confirmed by MALDI-TOF mass spectrometry using an AXIMA Confidence device (Shimadzu, Kyoto, Japan).

### Topical Administration of Tat-μCL

We performed topical instillation (20 μl) of 1 mM Tat-μCL dissolved in a sterile saline solution (pH 7.4) twice a day (9 a.m. and 5 p.m.) for 7 days in both eyes of adult female SD rats (3-month-old, weighing 350–400g) without anesthesia. The concentration and frequency of topical instillation of Tat-μCL were determined by the previous experiment using rhodopsin P23H transgenic rats [7]. Topical application was performed in home cages using a micropipette tip with residual liquid around the eyelids wiped off by a cotton swab. Every effort was taken to minimize suffering. Before the rat eyes were enucleated, rats were euthanized with inhalation of carbon dioxide. The peptide concentration was based on the findings of our previous study, that demonstrated topical instillation of 1 mM Tat-μCL eye drops resulted in photoreceptor protection without causing any adverse effects on the rhodopsin P23H (line 2) transgenic rats [7]. At each time point, 6 rats were examined, with 3 out of the 6 right eyes used for the immunohistochemistry and all of the left eyes used for the biochemical analyses. As a control, 3 rats were topically administered sterile saline for bilaterally using the same methods described above. Animals were randomly allocated into experimental or control group. Every effort was taken to reduce the number of animal used.

### Immunohistochemistry

Distribution of the peptide was analyzed by immune-positive reactions observed in and around the rat eyes. Immunohistochemical experiments were performed in line with our previously reported method [6]. Briefly, rats were euthanized after the final eye drop instillation by inhalation of carbon dioxide at 1, 3 and 6 h. Eyes were immediately enucleated after euthanasia. For the immunohistochemistry, eyes were fixed in 4% paraformaldehyde in phosphate-buffered saline (PBS), pH 7.4, for 20 min at room temperature. Eyes were then placed in the fixative overnight at 4°C. Specimens were cryoprotected for 4 h in 10% then 20% sucrose in PBS, and then frozen in OCT compound. Cryosections (5 μm thick) were made superior or inferior to the horizontal plane containing the optic disc and the central portion of the eye towards the equator. The sections were rinsed in PBS and blocked with 1% skim milk in PBS containing 0.05% Tween (PBS-T) for 2 h at room temperature. Sections were incubated overnight at 4°C with the mouse monoclonal antibody raised against HIV1 tat (ab63957, 1:200, Abcam, Cambridge, UK) diluted in 1% skim milk in PBS-T as the primary antibody. Sections were then washed with PBS-T and incubated with tetramethylrhodamine isothiocyanate isomer R (TRITC)-conjugated rabbit anti-mouse IgG (R0270, 1:200, Dako, Glostrup, Denmark) overnight at 4°C. Sections were washed with PBS-T and nuclei were counterstained with 4’ 6-diamidino-2-phenylindole (DAPI). Immunofluorescent images were obtained by laser confocal microscopy (FV1000-D; Olympus, Tokyo, Japan).

### Enzyme-Linked Immunosorbent Assay (ELISA)

The amount of the peptide delivered to the retina was quantified by sandwich ELISA as per a previously reported method [9]. In this procedure, we first added 50 μl of rabbit polyclonal anti-HIV1 tat antibody (1:2000, ab43014, Abcam) diluted with 0.05 M carbonate-bicarbonate buffer, pH 9.6, to 96-well immunoplates (Nunc, Roskilde, Denmark). After incubating the plates overnight at 4°C, the plates were washed three times with PBS-T, and then incubated with PBS-T containing 10% bovine serum albumin (BSA) at 37°C for 2 h. Subsequently, we then washed the plates with PBS and incubated the plates with 50 μl of retinal extract or standard peptide (100 amol, 1 fmol, 10 fmol, 100 fmol, 1 pmol, 10 pmol, 100 pmol and 1 nmol Tat-μCL) diluted with PBS-T containing 10% BSA at 37°C for 2 h. The retinal extract was obtained by sonication and gradual centrifugations at 600×g for 5 min, 4,500×g for 10 min, and 20,000×g for 20 min. The supernatant of the final centrifugation was used as the retinal extract. The protein concentration was measured by the Bradford’s assay with bovine serum albumin used as the standard. After washing the plate three times with PBS-T, the plates were incubated with 50 μl of mouse monoclonal anti-HIV1 tat antibody (1:1000, ab63957, Abcam) diluted with PBS-T containing 10% BSA at 37°C for 2 h. Subsequently, the plates were then washed three times with PBS-T and incubated with horse radish peroxidase (HRP)-conjugated rabbit anti-mouse IgG (1:1000) diluted with PBS-T containing 10% BSA at 37°C for 2 h. After incubation, the plates were washed three times with PBS-T and then once with distilled water. The reaction was performed by incubating the plates with 50 μl of citrate-phosphate buffer, pH 5.0, containing 0.04% o-phenylenediamine and 0.006% hydrogen peroxide. Color development was inhibited by the addition of 50 μl of 2.4 M sulfuric acid. Absorbance was determined at 492 nm.

### Statistical Analysis

All statistical analyses were conducted using the SPSS version 22 software program (IBM, Armonk, NY). A one-way ANOVA was used to statistically compare the results between multiple groups. And then, as a *post hoc* analysis, a Dunnett T3 test was used to determine significance between the two groups. In the present study, *P* < 0.05 was considered statistically significant. Experiments were performed in triplicate to confirm the reproducibility.

## Results

### Distribution and Kinetics of Topically Instilled Tat-μCL

**Fig 1:**
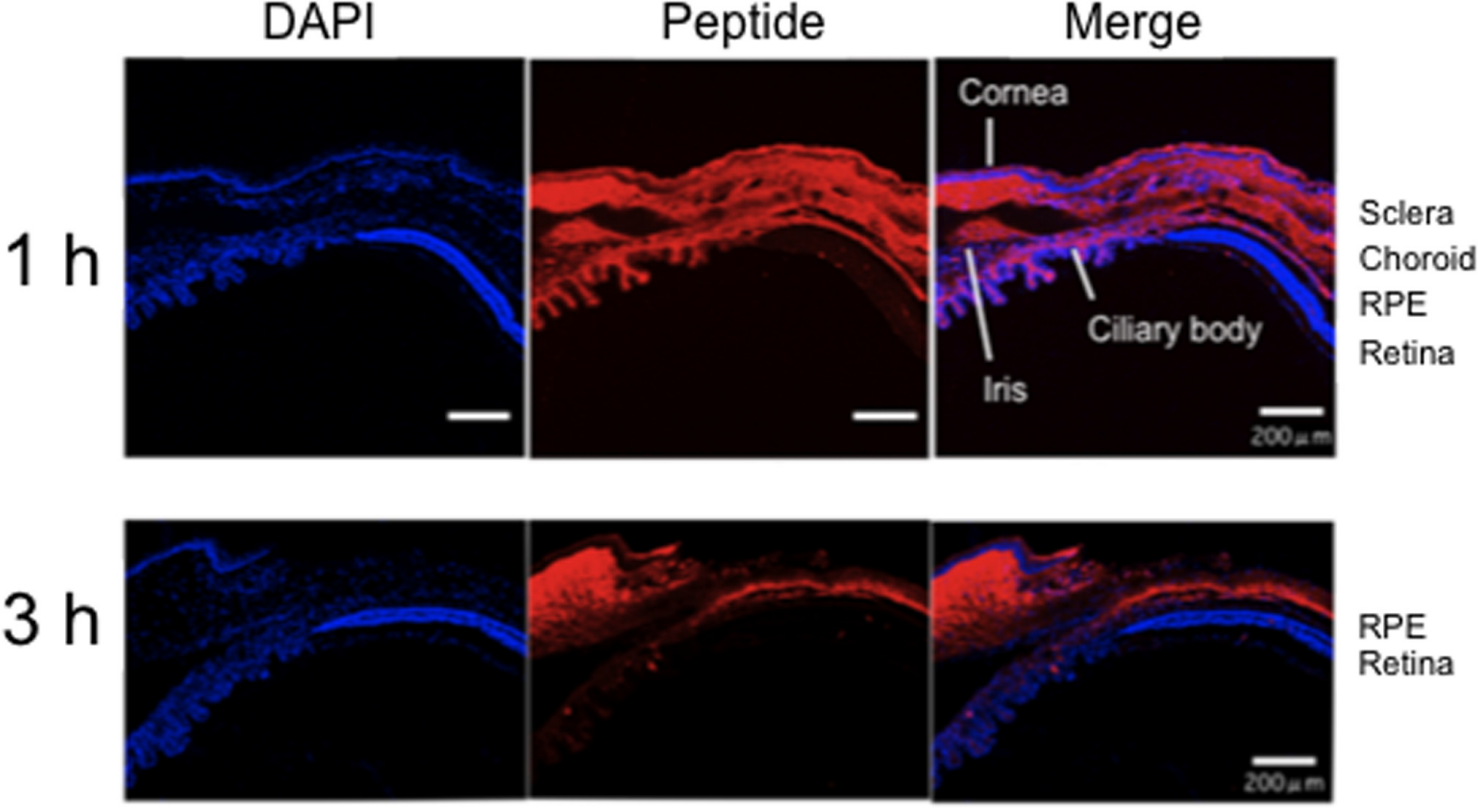
Distribution of topically instilled Tat-μCL in the anterior segment of the rat eye at 1 h (upper) and 3 h (lower) after the final eye drop application. Peptides are detected in the cornea, sclera, iris, ciliary body, choroid and RPE at the 1 h time point. The peptides also accumulated in the retina at the 1 h time point. The immunoreactivity weakened at the 3 h time point. DAPI: 4μ 6-diamidino-2-phenylindole. White bars indicate a 200 μm length. Results are representative of three independent experiments [n = 3 eyes (3 rats)].

All animal experiments were performed without any adverse effects. In the anterior segments, topically instilled Tat-μCL was diffusely distributed in the various tissues including the cornea, sclera, iris, ciliary body, choroid and retinal pigment epithelium (RPE) (Fig 1). The immunoreactivity was most prominent in these tissues at 1 h after the final application (Fig 1, upper panel) with a diminished immunoreactivity seen at 3 h after the final instillation (Fig 1, lower panel). The Tat-μCL was also positive in the neural retina, although the intensity was minimal even at 1 h (Fig 1).

**Fig 2:**
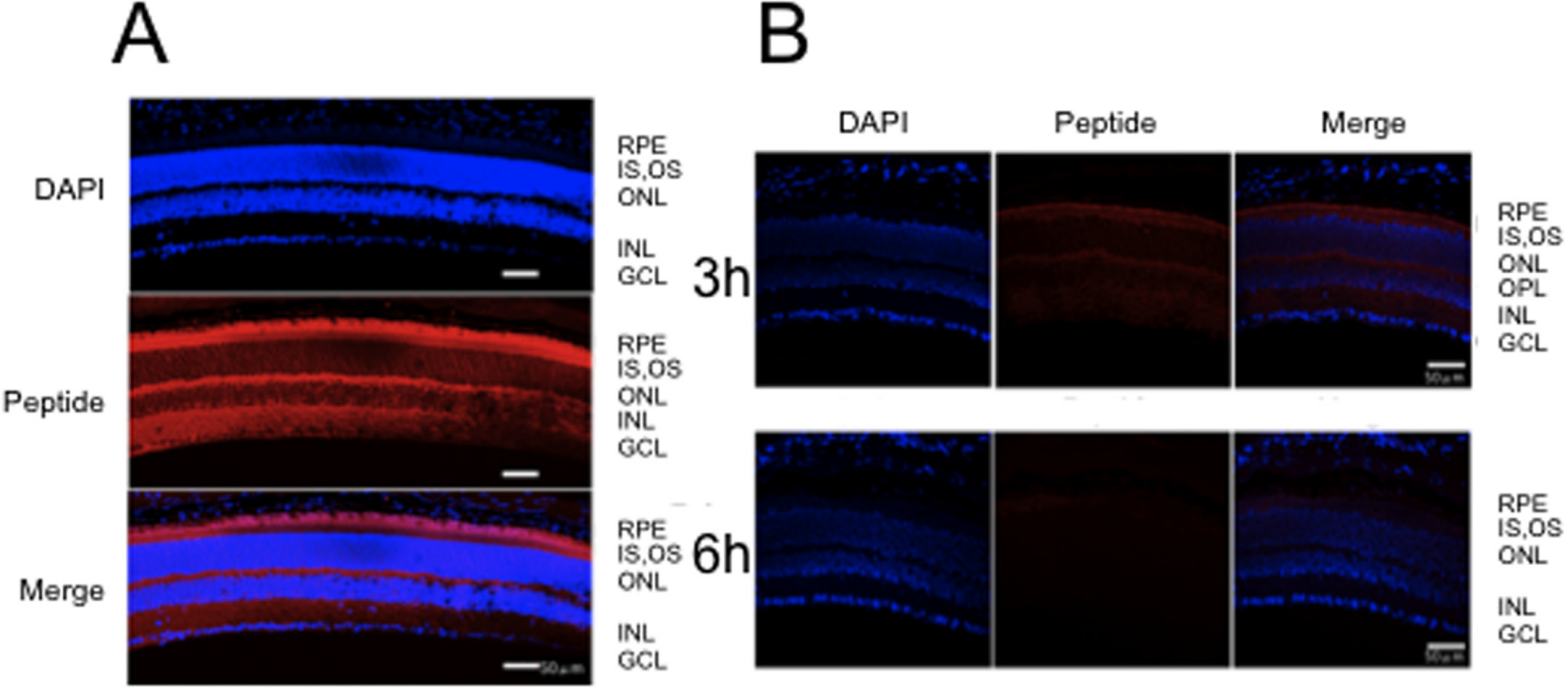
Distribution of topically instilled Tat-μCL in the posterior retina of the rat eye at 1, 3 and 6 h after the final eye drop application. A, Distribution of Tat-μCL at 1 h after the final eye drop application. Peptides can be detected in all layers of the retina including the photoreceptor layer and the ganglion cell layer. DAPI: 4’ 6-diamidino-2-phenylindole. White bars indicate a 50 μm length. Results are representative of three independent experiments [n = 3 eyes (3 rats)]. B, Distribution of Tat-μCL at 3 h (upper) and 6 h (lower) after the final eye drop application. There is a gradual disappearance of the peptides from the retina until 6 h after the application. DAPI: 4’ 6-diamidino-2-phenylindole. White bars indicate a 50 μm length. Results are representative of three independent experiments [n = 3 eyes (3 rats)].

The same tendency was observed in terms of intensity of the immunoreactivity in the posterior segments (Fig 2). The Tat-μCL was diffusely distributed throughout the retina, including the photoreceptor outer segments, outer nuclear layer, inner nuclear layer, and ganglion cell layer. Similar to the anterior segments, the immunoreactivity was most abundant at 1 h after the final eye drop application and this was followed by a gradual reduction at 3 and 6 h after the final application (Fig 2). Although the immunoreactivity level was low, a small amount of the peptide was still seen at 6 h after the instillation (Fig 2).

**Fig 3:**
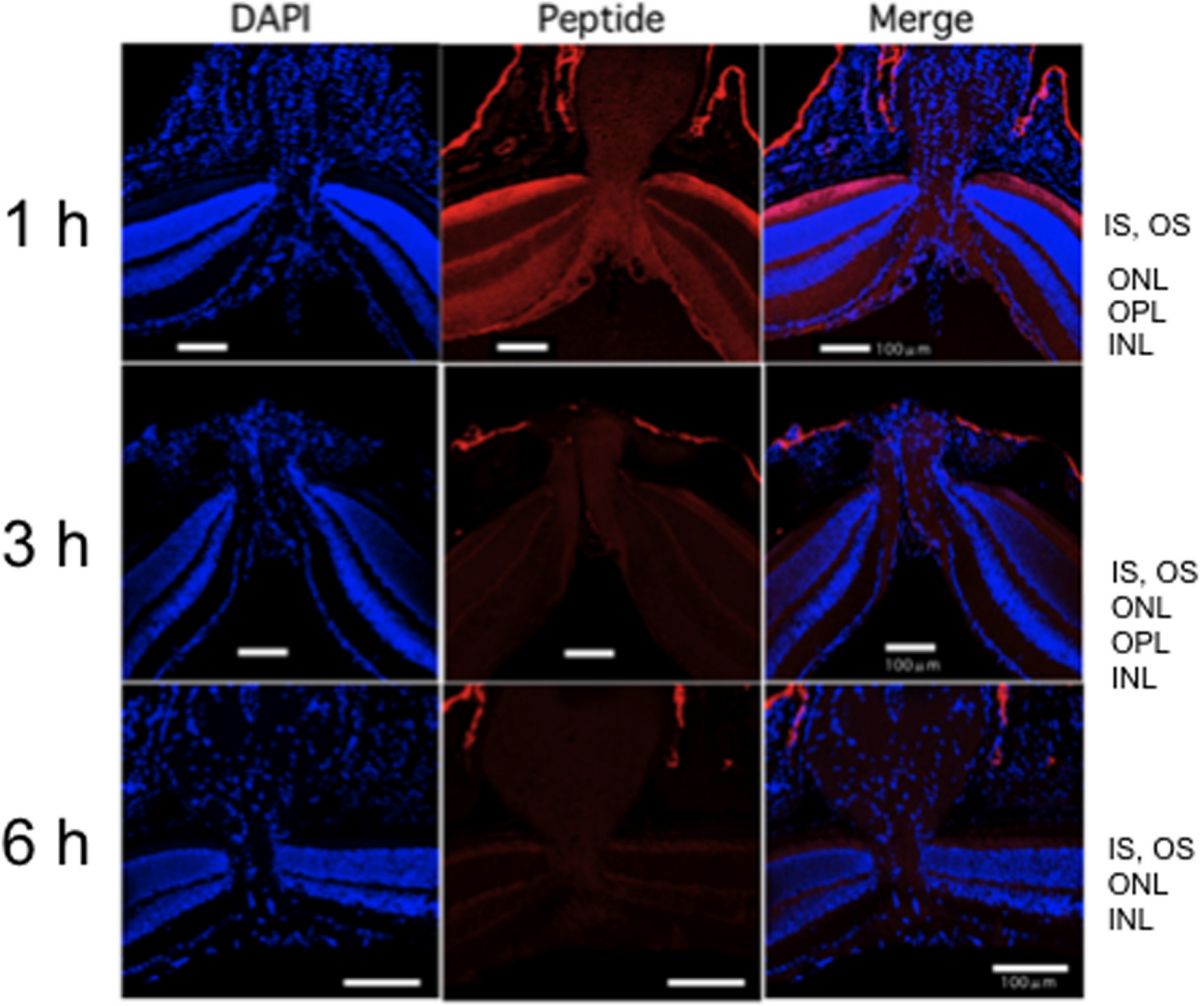
Distribution of topically instilled Tat-μCL in the optic disc area of the rat eye at 1, 3 and 6 h after the final eye drop application. Distribution of topically instilled Tat-μCL in the optic disc area of the rat eye at 1 h (upper), 3 h (middle), and 6 h (lower) after the final eye drop application. Peptides can be detected in the optic nerve, optic disc, all layers of the retina around the optic disc, and the retrobulbar tissue. The immunoreactivity is most prominent at the 1 h time point, with a gradual reduction until the 6 h time point. White bars indicate a 100 μm length. Results are representative of three independent experiments [n = 3 eyes (3 rats)].

In the optic disc area, the immunoreactive Tat-μCL was detected in the optic nerve, optic nerve sheath, optic disc, all layers of the retina, and the retrobulbar connective tissues (Fig 3). The same tendencies found for the other ocular tissues were also observed in terms of the time course. The immunoreactivity was most prevalent at 1 h after the final instillation, with the immunoreactivity gradually diminishing at the 3 and 6 h time points (Fig 3). Similar to the posterior segment, a small amount of the peptide remained in the retina at 6 h after the final application (Fig 3). With regard to the distribution of the peptide in the retina, there was a stronger immunoreactivity in the photoreceptor outer segment versus that seen in the inner plexiform layer (Figs 2 and 3).

### Quantification of the Tat-μCL

**Fig 4:**
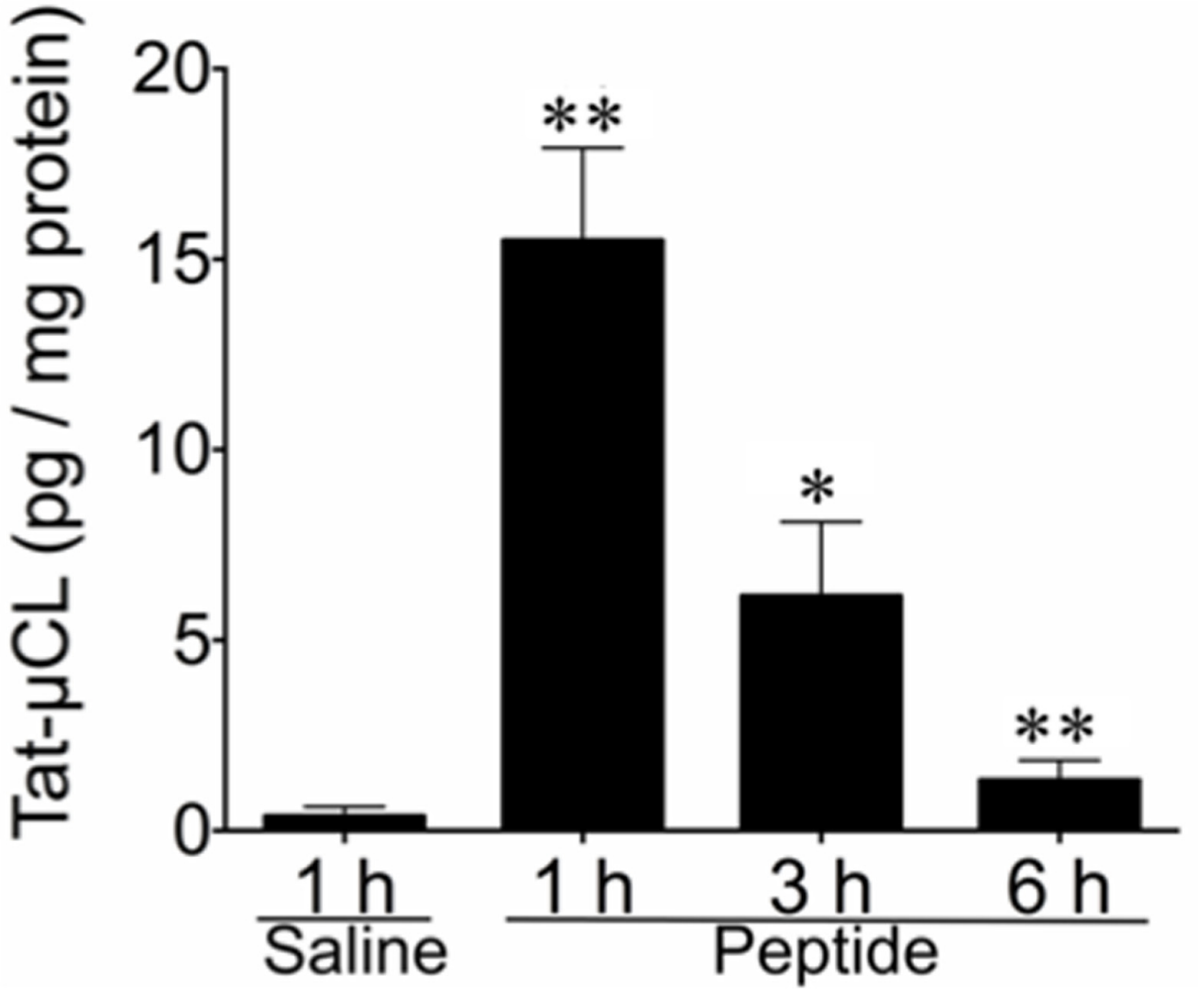
Quantitative analysis of topically instilled Tat-μCL in the whole retina at 1 h, 3 h and 6 h after the final eye drop application. The amount of the peptide was determined by a sandwich ELISA. Data are expressed as means ± standard deviation [n = 6 eyes (6 rats) per group]. **P < 0.001, *P = 0.001; each Tat-μCL-treated group versus saline-treated one (Dunnett T3 *post hoc* test).

Tat-μCL concentrations in the retina were quantitatively measured at each time point after the final eye drop using the sandwich ELISA procedure. We estimated the concentration of the Tat-μCL in the retina on average ± standard deviation to be about 15.3 ± 2.2, 5.8 ± 1.8 and 1.0 ± 0.4 pg/μg protein at 1, 3 and 6 h after the final instillation, respectively (Fig 4). Values at these three time points were significantly larger than the values observed for the control (*P* < 0.001 for ANOVA; *P* < 0.001 for 1 and 6 h, *P* = 0.001 for 3 h for Dunnett T3-test, respectively; raw data are presented in S1 Table). The ELISA results quantitatively proved the immunohistochemistry results (Figs 1–3).

## Discussion

Calpains are members of the Ca^2+^-activated cysteine proteinases and play important roles in various physiological and pathological pathways, such as regulating signal transductions, the cell cycle, cell adhesion, gene expression, and cell death [10]. Although calpains have long been believed to be cytosolic enzymes, they have also been shown to be present in the mitochondria as well as in the cytosol [11–13]. Previously, we demonstrated that the mitochondrial calpains are activated during photoreceptor degeneration in RCS rats in association with the nuclear transfer of the apoptosis-inducing factor (AIF) from the mitochondria [8]. The findings of our previous study also demonstrated that inhibition of the mitochondrial calpains could suppress photoreceptor cell death by down-regulation of the AIF transfer to the nucleus. This indicated that mitochondrial calpains could be targeted and used to develop potential treatments of inherited retinal degenerations. Basic biochemical differences exist for the μ- and m-calpains between the cytosol and mitochondria. For example, active mitochondrial calpains form heterotrimers consisting of large and small fragments along with the molecular chaperones ERp57 for μ-calpain and Grp75 for m-calpain [14, 15], while cytosolic μ- and m-calpains form heterodimers without any chaperones [9]. Based on these chemical properties, we developed a novel peptide that specifically inhibits the mitochondrial μ-calpain by acting as a decoy that blocks the binding between the large fragment and ERp57 [6]. Because cytosolic calpains have been known to play important roles not only in cell death but also cell survival, there is a risk that inhibition of the entire cytosolic calpains could cause deterioration of physiological cell functions. Moreover, the long-term inhibition of cytosolic μ-calpain has been shown to lead to the development of dilated cardiomyopathy due to the accumulation of abnormal protein aggregates. These findings suggest that cytosolic μ-calpain appears to play a role in the proteasomal protein degradation [16]. Therefore, specific inhibition of the mitochondrial calpains might be more appropriate for use in protecting photoreceptors from programmed cell death in hereditary retinal degeneration cases like retinitis pigmentosa. Although it has been reported that an inhibitory peptide against the mitochondrial m-calpain induced structural and functional impairments of the retinal tissues [17], our previous studies successfully demonstrated that even with topical instillation, Tat-μCL retarded the photoreceptor degeneration in RCS and rhodopsin S334ter or P23H transgenic rats [6, 7]. In the present study, we attempted to determine the distribution and kinetics of topically instilled Tat-μCL in the rat ocular tissues. Since retinitis pigmentosa is a chronic progressive disease, the photoreceptor protection treatments in these patients need to be continued for a long duration. Therefore, development of a minimally invasive therapy such as topical application is highly desirable. Furthermore, detailed information regarding the distribution and kinetics of the peptide is necessary in order to design a suitable topical instillation protocol in the future.

The results obtained in the present study revealed that the immunoreactive Tat-μCL was able to reach the retina in the posterior segment and the optic nerve within 1 h after topical application. Furthermore, the peptide did not remain in the ocular tissues, with disappearance of the majority of the peptide by 6 h after the final application. Although this turnover time was much shorter than that previously reported for nerve growth factor (26 kDa), which showed a peak of 6 h in the retina after eye drop application [3], the results were compatible with topically applied insulin (5 kDa), which exhibited a peak level at 1 h in the retina and 20 min in the optic nerve after instillation [2]. Because of its relatively small molecular weight (2.9 kDa), it is reasonable to assume that Tat-μCL is rapidly degraded by the proteolytic activities in the tissue, even though it is immediately distributed throughout the retina and optic disc area. Because there was such a rapid degradation in our present study, it is understandable why a topical treatment lasting 7 days would be viewed as being unnecessary. However, because retinitis pigmentosa is a chronic disease that requires clinical practices to administer long-term treatments, this study was designed to examine the distribution of the instilled peptide in the eye during consecutive eye drop treatments. Therefore, we analyzed the distribution of the peptide after 7 days of topical treatments.

There are two possible routes that the peptide could follow when traveling to the retina and optic disc area. In order to reach the retina and optic nerve, the first route would require that the peptide pass through the conjunctiva, anterior sclera, ciliary body, and vitreous humor, while in the second route the peptide would have to pass through the conjunctiva, periocular connective tissue, anterior and posterior sclera / optic nerve sheath, choroid, and RPE. Our present results revealed that the intensity of the immunoreactivity was stronger in the outer layer of the retina than in the inner layer, which suggests that the peptide was absorbed by the sclera from the periocular tissue, thereby immediately reaching the retina and optic nerve from the outside of the eyeball.

Based on our present ELISA assay, we estimated the concentration of the peptide in the retinal extract on average to be about 15.3, 5.8 and 1.0 pg/mg protein at 1, 3 and 6 h after the final eye drops, respectively. These values are roughly equivalent to the presence of 4.48, 1.70 and 0.29 μM of the peptide in the intracellular space at each of the respective time points. Because the value of 0.29 μM at 6 h after the final instillation is sufficiently higher than the IC50 (197 nM [6]), it is theoretically possible that the estimated amounts of the Tat-μCL that accumulated in the retina and optic nerve area were large enough to cause inhibition of the mitochondrial μ-calpain for up to at least 6 h after the final application. In addition, according to the results of ELISA assay (Fig 4), the half-life of Tat-μCL is estimated to be approximately 2.6 h in the rat retina. This estimation indicates that the concentration of Tat-μCL would reach the level of the IC50 roughly between 7 and 8 h after the instillation. Therefore, to keep the concentration of Tat-μCL in the rat retina above the IC50 to maintain the minimal therapeutic effect, the frequency of instillation of 1 mM Tat-μCL would be every 6 h for rats. But, it would depend on the initial concentration of the peptide, and that needs to be analyzed in the next step.

The limitations of our present study include 1) the immunoreactivity against a Tat-domain does not necessarily indicate that it represented the intact Tat-μCL peptide, as the possibility exists that partially degraded peptides could have been involved; and 2) since the accumulation ratio of the peptide can differ between wild-type and dystrophic rat eyes, the possibility exists that the pharmacokinetics of Tat-μCL may not be the same in the dystrophic rat retinas. The impact of these limitations will need to be clarified in a future study. However, if the immunoreactivities that we detected in our present experiments were indeed due to the intact Tat-μCL peptide, then the present findings suggest that 1 mM Tat-μCL eye drops need to be instilled in adult rat eyes at least every 6 h per day in order to maintain a therapeutic level of the peptide in the retina. This speculation appears to be supported by the results of our previous studies that showed that topically instilled peptide resulted in photoreceptor protective activity in three distinct rat models of retinal dystrophy [6, 7]. Moreover, we will also need to determine if the delivery of the peptide to the retina in other species of animals such as rabbits is similar to our present findings in the rat eyes. Additional investigations that clarify the questions raised in the present study will need to be performed in the future.

## Supporting Information

S1 Checklist The ARRIVE Guidelines Checklist. (PDF)

S1 Table Raw data for Fig 4. Each individual data of ELISA assay to measure the concentration of Tat-μCL in the retinal extract (pg/mg protein). Abbreviations; A, physiologic saline; B, C, D, Tat-μCL groups, respectively.
(PDF)
