# Supplementary figures and images for: Delivery of Topically Applied Calpain Inhibitory Peptide to the Posterior Segment of the Rat Eye
Source: PLoS One. 2015 Jun 24;10(6):e0130986. doi: 10.1371/journal.pone.0130986 (PMC4479448; doi:10.1371/journal.pone.0130986)

Supporting Table: Experimental Data

| 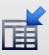 | Group A | Group B | Group C | Group D |
|----------------------------------------------------------------------------------|---------|---------|---------|---------|
|                                                                                  | 1 h     | 1 h     | 3 h     | 6 h     |
|                                                                                  | Y       | Y       | Y       | Y       |
| 1                                                                                | 0.2     | 14      | 7.6     | 1.3     |
| 2                                                                                | 0.4     | 16      | 5.4     | 1.7     |
| 3                                                                                | 0.5     | 15      | 8.5     | 1.4     |
| 4                                                                                | 0.3     | 19      | 4.5     | 1.2     |
| 5                                                                                | 0.1     | 12      | 5.6     | 1.6     |
| 6                                                                                | 0.8     | 17      | 5.4     | 1.0     |

Supplement: S1 Table — Each individual data of ELISA assay to measure the concentration of Tat-μCL in the retinal extract (pg/mg protein). Abbreviations; A, physiologic saline; B, C, D, Tat-μCL groups, respectively. (PDF) [file pone.0130986.s002.pdf]
